# Detecting Cognitive Impairment in African American Older Adults Using the LASSI-L and Plasma P-Tau_217_

**DOI:** 10.4236/aad.2025.142002

**Published:** 2025-06-30

**Authors:** Kirsten Horne Crenshaw, Alexandra Ortega, Rosie E. Curiel Cid, Diane D. Zheng, Minerva M. Carrasquillo, Elizabeth Crocco, Sofia Ramirez, Alexia Frydman, Stephanie Remedios, Yariannis Vazquez Morales, David E. Vaillancourt, Wei-En Wang, David Fernandez Garcia, Juan Pablo de Rivero Vaccari, Nilüfer Ertekin-Taner, Lindsey Kuchenbecker, Ranjan Duara, David A. Loewenstein

**Affiliations:** 1 Center for Cognitive Neuroscience and Aging (CNSA), Department of Psychiatry and Behavioral Sciences, Miller School of Medicine, University of Miami, Miami, FL, USA; 2 Florida Alzheimer’s Disease Research Center (ADRC), University of Florida, Gainesville, FL, USA; 3 Department of Neurology, Mayo Clinic, Jacksonville, FL, USA; 4 Department of Applied Physiology and Kinesiology, University of Florida, Gainesville, FL, USA; 5 Department of Neurological Surgery and The Miami Project to Cure Paralysis, Miller School of Medicine, University of Miami, Miami, FL, USA; 6 Mount Sinai Medical Center, Miami Beach, FL, USA

**Keywords:** Alzheimer’s Disease, Mild Cognitive Impairment, P-Tau217, LASSI-L

## Abstract

**Background:**

Alzheimer’s disease (AD) disproportionately affects Black/African American (B/AA) older adults, yet this group remains underrepresented in research. Traditional neuropsychological assessments, often developed on predominantly White populations, may not be reliable for B/AA individuals. The Loewenstein-Acevedo Scales for Semantic Interference and Learning (LASSI-L) have been shown to effectively differentiate individuals with amnestic mild cognitive impairment (aMCI) from cognitively unimpaired (CU) individuals. This study examines the relationship between LASSI-L performance and plasma p-tau_217_ levels to explore early detection methods for AD in B/AA populations.

**Methods:**

Fifty-six older adults received clinical and cognitive evaluations and were deemed cognitively unimpaired (CU) and p-tau_217_ negative (n = 35) or met criteria for amnestic mild cognitive impairment (aMCI) and p-tau_217_ positive (n = 21). All participants were administered the LASSI-L to compare groups, but it was not used for group allocation to avoid circularity.

**Results:**

After adjusting for age and MMSE score, the aMCI p-tau_217_+ group performed significantly worse than the CU p-tau_217_− group on both free recall on List B (Free B1 Recall) and frPSI (correct responses on Cued B2). These differences remained statistically significant after covariate adjustment (p < 0.001). In addition, four other outcomes remained statistically significant following covariate adjustment: the aMCI p-tau_217_+ group exhibited a higher percentage of intrusion errors (PIE) on both Cued B1 and Cued B2, along with poorer performance on maximal learning ability (Cued A2) and PSI (correct responses on Cued B1). However, after applying the Bonferroni correction, only PIE on Cued B2 remained statistically significant among these measures. Notably, performance on LASSI-L Free B1 Recall and PIE for List Cued B2 were significant predictors distinguishing aMCI p-tau_217_+ from CU p-tau_217_− groups, with high sensitivity (80%) and specificity (91.7%). ROC analysis of these predictors yielded an area under the curve of 0.872 (SE = 0.055; p < 0.001), with a 95% confidence interval ranging from 0.765 to 0.979.

**Conclusion:**

The study highlights the utility of the LASSI-L in conjunction with plasma biomarkers, particularly p-tau_217_, for early AD detection in B/AA older adults. The LASSI-L demonstrated strong sensitivity to cognitive impairment, effectively differentiating between CU and aMCI groups based on plasma p-tau_217_ levels. These findings suggest that combining cognitive assessments with plasma biomarkers can enhance early diagnosis and improve timely interventions, addressing health disparities in AD diagnosis and care.

## 1. Introduction

Alzheimer’s disease (AD) is one of the most prevalent conditions impacting older adults, with Black/African American (B/AA) older adults being disproportionately impacted. Specifically, among B/AA older adults, approximately 20% are living with AD, nearly double the prevalence seen in their white counterparts (11.5%), despite often experiencing delayed or missed diagnoses altogether [1]-[3]. Despite being heavily impacted, B/AA older adults remain one of the most underrepresented groups in AD research [2]. This underrepresentation poses a critical issue, especially since many neuropsychological assessments were developed and normed primarily on White populations, often failing to account for cultural differences. As a result, these assessments may lead to inaccurate diagnoses for B/AA individuals. The lack of reliable and culturally relevant assessments for B/AA older adults is particularly troubling given that existing literature highlights lower baseline global cognition, poorer performance on cognitive assessments, and a slower rate of cognitive decline associated with AD [4] [5]. This further emphasizes the need for more accurate and culturally sensitive assessments for B/AA older adults to ensure these individuals can receive timely and appropriate interventions.

Although traditional neuropsychological assessments may lack reliability in B/AA populations, the Loewenstein-Acevedo Scales for Semantic Interference and Learning (LASSI-L) is an innovative cognitive challenge test that is sensitive to early cognitive changes in preclinical and prodromal AD and other neurodegenerative conditions [6]-[10], while also being culturally fair and effective at differentiating individuals with amnestic mild cognitive impairment (aMCI) from cognitively unimpaired (CU) individuals across a number of ethnic and cultural groups including B/AA populations which is an outcome not consistently achieved with traditional neuropsychological assessments [11]-[13].

In addition to these novel cognitive assessments, advances in plasma-based biomarkers, particularly p-tau_217_, demonstrate excellent promise for the early detection of AD [14]. P-tau_217_ consistently demonstrates high sensitivity and specificity to Aβ PET positivity and tau PET results [15] [16]. Research has shown that p-tau_217_ levels correlate with both current AD brain pathology and future cognitive performance. Additionally, a recent study with plasma p-tau_217_ has indicated that this measure is equally effective as amyloid PET imaging in identifying various stages of AD progression [17]. These findings suggest p-tau_217_ may serve as a strong predictor for detecting AD even before clinical symptoms appear and may serve as a valuable indicator during the different stages of AD [18]. There is increasing interest in the use of plasma-based biomarkers like p-tau_217_ which has been heralded as an extremely promising approach to use for screening purposes in primary care settings. However, a limitation of this approach is that abnormal amyloid accumulation may occur many years before clinical symptoms [19]-[21]. Thus, elevated plasma p-tau_217_ does not necessarily mean that cognitive or functional impairment is present or even unfolding. To that end, blood-based biomarkers in the absence of sensitive cognitive measures may not have clinical relevance in primary care or community health settings. Of particular concern is the underrepresentation of B/AA older adults in much of the rapidly evolving research related to plasma biomarkers, as well as their decreased likelihood of seeking cognitive evaluations despite having twice the prevalence of AD and AD-Related Dementias (ADRD) relative to the general population [22]-[25].

The LASSI-L has been found to be related to PET amyloid burden as well as elevated levels of plasma p-tau_181_ and A*β* 42/40 [26]-[29] and has shown good generalizability to older Hispanic and non-Hispanic populations. Unfortunately, the LASSI-L has not yet been related to p-tau_217_ or any other plasma biomarkers of AD in a B/AA cohort. Therefore, the current investigation represents the first systematic attempt to examine differences in LASSI-L performance among cognitively impaired B/AA older adults with suspected underlying AD pathology (elevated plasma p-tau_217_) and CU B/AA older adults without elevated plasma p-tau_217_. Based on previous research, it was hypothesized that SIEs and other cognitive deficits measured by the LASSI-L could discriminate between B/AAs who were cognitively impaired likely due to underlying AD and controls.

## 2. Methods

### 2.1. Study Population

Fifty-six participants aged 60 and above from two IRB-approved studies with identical clinical and neuropsychological profiles were included from the following two studies: Florida Alzheimer’s Disease Research Center (ADRC) and Innovative Deep Phenotyping of African Americans at Risk for Alzheimer’s disease. Both studies were IRB approved (*i.e.*, AG047726602/ AG066506 and AG077677).

All participants were community dwellers who were independent in completing basic and instrumental activities of daily living and did not meet DSM-5-TR criteria for Major Neurocognitive Disorder or any other significant neuropsychiatric disorder that could preclude reliable cognitive assessments [30].

The standardized protocol consisted of neuropsychological battery and annual clinical assessment protocol which was administered at the same time the Hopkins Verbal Learning Test-Revised [31], a measure of episodic memory including a single paragraph using recall of the passage [32], Category Fluency [33], Stroop Color Word Test [34], and Trail Making Test Part A and B [35]. The neuropsychological assessments and clinical interviews were completed separately by experienced clinicians to avoid criterion contamination. All participants were evaluated in English. Diagnostic determination was made by a multidisciplinary consensus panel and independent of the LASSI-L to avoid diagnostic circularity.

### 2.2. Cut-Offs for p-tau_217_ Positivity

Venous blood was collected using 10mL Purple Top tubes containing EDTA as an anticoagulant, mixed by inversion ten times, and centrifuged at room temperature for 12 minutes at 1700 RPM within one hour of collection. Aliquots of 500 microliters of plasma were then frozen and stored at −80 degrees Celsius (C). Prior to analysis, the samples were thawed (1 freeze-thaw cycle) at room temperature, vortexed for 30 seconds, and placed on ice until centrifuging at 10,000g for five minutes at 4C. Single molecule array (SIMOA) technology for p-tau_217_ (ALZpath; Quanterix, Billerica, MA) was employed. Duplicate samples were analyzed. We only included samples with a coefficient of variation (CV) 20% or less. It is common practice in our laboratories and others to exclude p-tau_217_ values that have a duplicate covariation of greater than 20%. This is because the reliability of the findings with such a high coefficient of variability is unreliable.

Internally derived cutoffs for “positive” plasma p-tau_217_ were based on correspondence with the visual read of an A*β*-PET scan contiguous with the blood draw from our ADRC (N = 239). A cutoff value of > 0.55 pg/ml representing a positive plasma p-tau_217_ value was derived from the receiver operating characteristic (ROC) curve and Youden’s index. The cutoff value of 0.55 pg/ml, representing a “positive” plasma p-tau_217_ test had a sensitivity of 89% and a specificity of 82% [36]. In a series of several datasets across the globe, Ashton and colleagues [14] found a correspondence with amyloid PET scans at the 95th percentile at plasma p-tau_217_ > 0.63 pg/ml, but acknowledged that p-tau_217_ scores at our cut-off of > 0.55 pg/ml likely reflected AD pathology accumulation. As such, our cut off of > 0.55 pg/ml was maintained to represent amyloid positivity. However, Ashton and colleagues [14] argued that p-tau_217_ as low as 0.43-0.55 pg/ml may still represent amyloid accumulation. Thus, to be conservative, we employed Ashton’s cut-off of < 0.40 pg/ml as representing the absence of significant abnormal plasma amyloid accumulation.

### 2.3. Diagnostic Classification

Based on the participants comprehensive clinical interview and performance on the neuropsychological testing battery, diagnostic groups were classified utilizing the following criteria:

### 2.4. Cognitively Unimpaired p-tau_217_ Negative Group (CU p-tau_217_−; n = 35)

Participants were classified as CU p-tau_217_− if the following criteria were met: a) no subjective cognitive complaints by the participant and/or informant; b) Global Clinical Dementia Scale (CDR) of 0; c) performance across measures demonstrated scores less than 1.0 SD below the mean accounting for age and education, d) P-tau_217_ was deemed negative if below the p-tau_217_ cutoff of < 0.40 pg/ml as described above.

### 2.5. Amnestic Mild Cognitive Impairment p-tau_217_ Positive Group (aMCI p-tau_217_+; n = 21)

Participants were classified as aMCI p-tau_217_+ if the following criteria were met: a) subjective cognitive complaints by the participant and/or informant; b) Global CDR of 0.5; c) one or more memory measures on the standardized battery listed above demonstrated scores at least 1.5 SD or more below the mean accounting for age, education, and language; d) p-tau_217_ was deemed positive if above the p-tau_217_ cutoff of > 0.55 pg/ml.

### 2.6. Loewenstein-Acevedo Scales for Semantic Interference and Learning (LASSI-L)

All participants were administered the LASSI-L; however, it was not utilized in diagnosis to avoid circularity. The LASSI-L is a well-established cognitive stress test validated in both English and multiple other languages. It has demonstrated strong test-retest reliability as well as high discriminative and concurrent validity [11] [37]-[40]. Furthermore, a preliminary study with the LASSI-L suggested it is culturally fair and effective in distinguishing individuals with aMCI from CU individuals in B/AA populations [13].

During administration of the LASSI-L, the participant uses controlled learning and cued recall to maximize storage of an initial list of 15 target words (List A) representing one of the three semantic categories (*i.e.*, fruits, clothing, musical instruments). After the 15 words from List A have been presented, there is a free recall trial and then cued recall trials for each of the three categories consisting of five words per category. List A is presented again along with the cued recall trials for each of the three categories. Afterwards, a second list of different words for free recall (List B) from the same semantic categories is presented immediately after the second trial of List A. The presence of shared semantic categories between Lists A and B leads to a significant amount of proactive semantic interference (PSI), which is measured during Cued B (Cued B1 recall). Compared to conventional memory assessments, the re-presentation and subsequent recall of this second word list evaluates an individual’s capacity to recover from the effects of PSI (frPSI) (Cued B2 recall). Of note, the participant is later provided with category cues to determine how many words they are able to recall from List A to measure retroactive semantic interference and the later recovery from PSI is not assessed by any other memory paradigm. For more information regarding LASSI-L, refer to Curiel and colleagues [37] [38].

In the current study, we examined the LASSI-L variables including maximal learning ability (Cued A2), PSI (correct responses on Cued B1), frPSI (correct responses on Cued B2), and semantic intrusion errors (SIE) that occurred during both Cued B1 and Cued B2. To assess SIEs, a method was developed using the percentage of intrusion errors (PIE) relative to total correct responses, accounting for the relationship between errors and total responses which provides more information compared to the absolute number of intrusions alone which is calculated by the formula: Intrusion Errors/(Intrusion Errors + Correct Responses) [41]. PIE was utilized in the current study and has previously demonstrated to be highly sensitive to early cognitive impairment [41] [42].

## 3. Statistical Analyses

For initial comparisons between diagnostic groups, a one-way analysis of variance (ANOVA) and analysis of covariance (ANCOVAs) controlling for age and performance on the MMSE were employed. Based on obtained findings, a stepwise binary logistic regression analysis was performed to determine the extent to which LASSI-L variables could distinguish between CU p-tau_217_− and aMCI p-tau_217_+ groups. Afterwards, a receiver operative characteristic (ROC) analysis was conducted to examine the sensitivity and specificity in the LASSI-L variables’ ability to differentiate between groups.

## 4. Results

As demonstrated in Table 1, the CU p-tau_217_− and aMCI p-tau_217_+ groups were found to have similar educational backgrounds and similar gender distribution with no statistically significant differences. Age and performance on the MMSE, a global cognitive screener, were statistically significant indicating those who are aMCI p-tau_217_+ were older and presented with worsening performance on the MMSE compared to those who were deemed CU p-tau_217_. Performance across the LASSI-L demonstrated B/AA aMCI p-tau_217_+ participants presented with worsening performance compared to the CU p-tau_217_− participants across all LASSI-L variables. After controlling for age and performance on the MMSE, performance on the LASSI-L subtests across aMCI p-tau_217_+ and CU p-tau_217_− were subsequently examined.

An inspection of Table 2 after adjustment for covariates allowed for us to determine both changes in LASSI-L p-values and effect sizes could be compared to unadjusted values in Table 1 (all p< 0.002). Since the inclusion of covariates reduced degrees of freedom and statistical power, we first present unadjusted values since performing Bonferroni measures across so many tests would likely result in familywise type 2 errors (accepting a false null hypothesis). Performance across the LASSI-L demonstrated that B/AA aMCI p-tau_217_ + group performed significantly worse than the CU p-tau_217_− group on Free B1 Recall and Cued B2 Recall. These differences remained statistically significant after covariate adjustment (p< 0.001). In addition, four other outcomes remained statistically significant following covariate adjustment: the aMCI p-tau_217_+ group exhibited a higher percentage of intrusion errors on both Cued B1 and Cued B2, along with poorer performance on Cued A2 and Cued B1 Recall. However, after applying the Bonferroni correction, only PIE on Cued B2 remained statistically significant among these measures.

**Figure 1.**
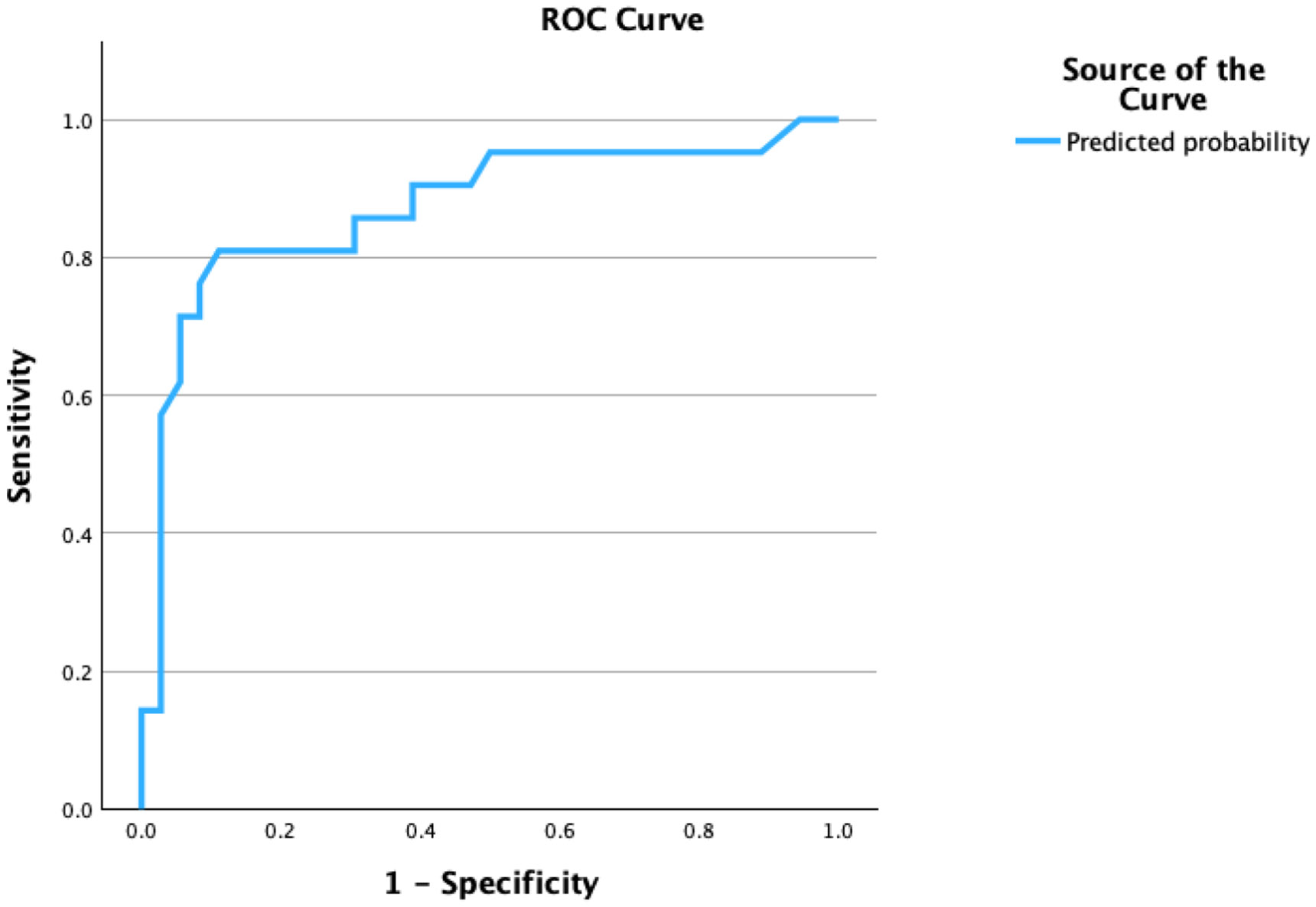
Area under the ROC curve.

Table 3 demonstrated performance on LASSI-L Free B1 Recall and PIE for List Cued B2 are significant predictors of aMCI p-tau_217_+ and able to distinguish between those who were aMCI p-tau_217_+ versus CU p-tau_217_− with high sensitivity (80%) and specificity (91.7%). These two variables yielded a single predicted probability score, which was then employed in ROC analysis which yielded an area under the curve of 0.872 (SE = 0.055); p < 0.001. At the 95th percentile, the lower bound was 0.765 while the upper bound was 0.079 (Figure 1).

## 5. Discussion

This is the first study to examine differences in LASSI-L performance between aMCI B/AA older adults with suspected underlying AD pathology (high p-tau_217_ load) and CU B/AA older adults without suspicion of underlying AD (low p-tau_217_ load). Results demonstrated that LASSI-L variables, specifically PIE for Cued B2, Free B1 Recall, and Cued B2 Recall were able to effectively discriminate between aMCI and CU B/AA groups, and that the LASSI-L could distinguish between groups with differing p-tau_217_ levels. This study highlights the utilization of the LASSI-L in combination with plasma biomarkers to enhance early detection of AD/ADRD, marking a significant finding as this is the first time the LASSI-L correct recall responses and SIEs have been linked to p-tau_217_ in a B/AA cohort. This suggests that the LASSI-L cognitive challenge test is indicative of cognitive deficits which may reflect difficulties in the participant’s ability to maintain accurate memory and an increased vulnerability to inaccurate recall. This finding aligns with previous literature showing that the LASSI-L could be a useful predictor of clinical progression over time in at-risk older adults since the LASSI-L was able to accurately depict those patients who progressed from Pre-MCI to aMCI [42].

As expected, the aMCI p-tau_217_+ group exhibited inferior performance on free recall across PSI (correct responses on Cued B1) and frPSI (correct responses on Cued B2). These findings suggest the LASSI-L remains sensitive to cognitive changes and can effectively discriminate between B/AA older adults who are cognitively impaired versus CU. This aligns with previous studies [29] [41] [42], highlighting the potential of the LASSI-L as a reliable tool for assessing cognitive function in B/AA populations and underscores the importance of utilizing cognitive assessments in early detection. While plasma biomarkers, particularly p-tau_217_, have shown sensitivity and predictive value for AD, these may be elevated in the absence of clinical symptoms. Thus, for clinical screening and evaluation, these exciting emerging biomarkers can best be used in conjunction with cognitive assessments, such as the LASSI-L, as biomarkers alone do not indicate the presence of current cognitive impairment. Early access to plasma biomarkers, even before clinical symptoms emerge, may allow for timely referrals for cognitive evaluations, helping to assess the extent of cognitive impairment and inform appropriate interventions. These findings are particularly significant for the B/AA population, given the high prevalence of AD and their lower likelihood of seeking cognitive evaluations. A p-tau_217_ assay, combined with the brief LASSI-L (a shortened version of the full LASSI-L), could provide valuable information in primary care or remote community settings, where access to specialty memory disorder centers or amyloid/tau neuroimaging may be limited.

There are several strengths of the current study. The study design allowed for participants to be carefully diagnosed utilizing identical clinical and neuropsychological protocols to place individuals into CU and aMCI groups. The LASSI-L was also not utilized when diagnosing the CU and aMCI groups which is a strength as it avoided circularity. Additional strengths include adjustments being employed in statistical methods for age and performance on a global cognitive screener (*i.e.*, MMSE). While the relationship between plasma biomarkers and cognitive decline in B/AA populations remains underexplored, our study adds to a growing body of evidence supporting the use of culturally fair cognitive assessments and easily accessible biomarkers in these communities. This evidence is valuable as it not only bridges the gap in the literature but also provides practical and non-invasive methods for early detection of AD/ADRD which may help address disparities in AD diagnosis and care among this underrepresented population. Limitations of the current study include the modest total sample size of 56 participants. Increasing the participant pool would strengthen the generalizability of the findings, enabling a more thorough analysis and a more accurate reflection of the diverse characteristics within the population. Further studies with aMCI participants with low p-tau and different etiologies will also provide meaningful information that would be valuable for assessing B/AA populations.

Although beyond the scope of the current investigation, future research should aim to examine medical comorbidities that might be related to both cognitive impairment and trajectories of cognitive and functional decline longitudinally to further assess the LASSI-L’s predictive abilities. This will enhance our knowledge of early detection of AD/ADRD in our underserved B/AA older adult populations.

## Figures and Tables

**Table 1.** Diagnostic groups, demographic, lassi-l performance, and plasma biomarkers in B/AA older adults.

	Cognitively Unimpaired p−tau217− (n = 35)	aMCI p−tau217+ (n = 21)	F-or X2 Value	p-value	Eta Squared
Age	65.94 (SD = 5.1) (range 56 - 78)	69.81 (SD = 6.1) (range 56 - 81)	6.12	p = 0.016	0.100
Education	14.31 (SD = 2.7) (range = 10 - 20)	14.52 (SD = 3.0) (range = 11 - 20)	0.08	p = 0.78	NA
MMSE Score (range 23 - 30)	28.71 (SD = 1.5) (range = 24 - 30)	26.05 (SD = 2.5) (range = 21 - 30)	16.88	p < 0.001	0.235
Female (%)	62.9%	52.4%	0.24	p = 0.411	NA
P-tau217 (Range = 0.180 - 3.15)	0.225 (SD = 0.07) range (0.100 - 0.370 pg/ml)	1.01 (SD = 0.55) range (0.560 - 2.710 pg/ml)	73.40	p < 0.001	0.572
LASSI-L Cued A2 Recall	13.53 (SD = 1.6) (range = 8 - 15)	10.97 (SD = 2.4) (range = 6 - 15)	18.60	p < 0.001	0.253
LASSI-L Free B1 Recall	7.14 (SD = 0.24) Range(2 - 12)	3.76 (SD = 2.5) (range 0 - 10)	22.70	p < 0.001	0.292
LASSI-L Free B1 Intrusions	0.83 (SD = 1.1) Range(0 - 4)	1.95 (SD = 2.1) (range 0 - 6)	22.70	p < 0.001	0.292
LASSI-L Cued B1 Recall	7.26 (SD = 2.7) (range 1 - 12)	4.76 (SD = 2.5) (range 0 - 10)	14.11	p < 0.001	0.204
LASSI-L Cued B1 Intrusions	1.97 (SD = 2.1) (range 0 - 11)	4.05 (SD = 3.0) (range 0 - 0)	9.93	p = 0.002	0.153
LASSI-L Cued B2 Recall	10.97 (SD = 1.5) (range 3 - 14)	7.57 (SD = 2.6) (range 3 - 12)	23.29	p < 0.001	0.297
LASSI-L Cued B2 Intrusions	1.57 (SD = 1.7) (range 0 - 7)	3.38 (SD = 2.3) (range 0 - 8)	10.24	p = 0.002	0.157
Percentage Intrusion Errors (PIE Cued B1)	0.204 (SD = 0.20) (range 0 - 0.85)	0.468 (SD = 0.27) (range 0 - 1.00)	18.11	p < 0.001	0.251
Percentage Intrusion Errors (PIE Cued B2)	0.118 (SD = 0.13) (range 0 - 0.64)	0.295 (SD = 0.17) (range 0 - 0.57)	18.52	p < 0.001	0.252

**Table 2.** African American diagnostic groups demographic, LASSI-L indices and plasma biomarkers after adjustment for age and MMSE scores.

	Cognitively Unimpaired p-tau217− (n = 35)	aMCI p-tau217 + (n = 21)	F-or X2 Value	p-value	Partial Eta Squared
LASSI-L Cued A2 Recall	13.21 (SE = 0.34)	11.84 (SE = 0.46)	4.82	p = 0.033	8.5%
LASSI-L Free B1 Recall	7.00 (SE = 0.46)	4.00 (SE = 0.62)	12.67	p < 0.001	19.6%
LASSI-L Free B1 Intrusions	1.05 (SE = 0.26)	1.68 (SE = 0.36)	1.65	p = 0.205	3.1%
LASSI-L Cued B1 Recall	7.14 (SE = 0.49)	4.96 (SE = 0.67)	5.88	p = 0.019	10.2%
LASSI-L Cued B1 Intrusions	2.48 (SE = 0.42)	3.21 (SE = 0.57)	0.890	p = 0.350	1.7%
LASSI-L Cued B2 Recall	10.75 (SE = 0.47)	7.94 (SE = 0.64)	10.53	p < 0.001	16.8%
LASSI-L Cued B2 Intrusions	1.77 (SE = 0.36)	3.05 (SE = 0.48)	3.82	p = 0.056	6.8%
Percentage Intrusion Errors (PIE Cued B1)	0.243 (SE = 0.04)	0.401 (SE = 0.06)	4.45	p = 0.04	8.0%
Percentage Intrusion Errors (PIE Cued B2)	0.135 (SE = 0.03)	0.267 (SE = 0.04)	6.87	p = 0.011	11.7%

**Table 3.** Predicting group membership (CU p-tau_217_− vs. aMCI p-tau_217_+) Using performance on LASSI-L.

	B	S.E.	Wald	df	Sig.
Pie Cued B2	5.80	2.5	5.47	1	0.019
LASSI Free Recall B	−0.497	0.717	9.05	1	0.002
Constant	1.038	1.011	1.054	1	0.305

Note: Sensitivity of Joint Predictors is 80.0% and specificity is 97.1%.
